# Report of 5 novel mutations of the α-L-iduronidase gene and comparison of Korean mutations in relation with those of Japan or China in patients with mucopolysaccharidosis I

**DOI:** 10.1186/s12881-016-0319-x

**Published:** 2016-08-12

**Authors:** Min Jung Kwak, Rimm Huh, Jinsup Kim, Hyung-Doo Park, Sung Yoon Cho, Dong-Kyu Jin

**Affiliations:** 1 Department of Pediatrics, Pusan National University Hospital, Pusan National University School of Medicine, 179 Gudeok-ro, Seo-gu Busan, 602-739 Korea; 2 Department of Pediatrics, Samsung Medical Center, Sungkyunkwan University School of Medicine, 81 Irwon-ro, Gangnam-gu Seoul, 135-710 Korea; 3 Laboratory Medicine & Genetics, Samsung Medical Center, Sungkyunkwan University School of Medicine, 81 Irwon-ro, Gangnam-gu Seoul, 135-710 Korea

**Keywords:** α-L-iduronidase, Genotype, Mucopolysaccharidosis I, Mutation

## Abstract

**Background:**

Mucopolysaccharidosis I (MPS I) is an autosomal recessive lysosomal storage disorder caused by a lack of the lysosomal enzyme α-L-iduronidase (IDUA). To date, more than 200 *IDUA* mutations have been reported. However, only a few types of mutations are recurrent and the frequencies of mutations differ from country to country.

**Methods:**

We performed the *IDUA* mutation analysis in seven patients who were biochemically diagnosed with MPS I in the Department of Pediatrics, Samsung Medical Center, from 2009 to 2014. Here, we describe the results of the *IDUA* mutation analysis in seven patients with MPS I and the *IDUA* mutational spectrum in Korean patients with MPS I, including previous data.

**Results:**

The *IDUA* mutations were found in all 14 alleles of 7 patients, and 11 kinds of *IDUA* mutations were identified. The detected mutations were five missense mutations (p.A79V, p.L346R, p.T388K, p.P496R, and p.C577Y), two nonsense mutations (p.Y618* and p.R628*), two deletions (c.683delC and c.1591delC), one splice site mutation (c.972+1G>A), and one duplication (c.613_617dup). Among these, p.T388K, p.C577Y, c.683delC, c.1591delC, and c.972+1G>A were novel mutations that have not previously been reported. After taking everything into consideration, including *IDUA* mutation analysis of the previously reported 10 unrelated Korean patients with MPS I, p.L346R and c.704ins5 were most commonly found in Korean patients with MPS I. However, p.W402* and p.Q70*, which have mainly been found in Caucasian patients, were not found.

**Conclusion:**

As a result, p.L346R and c.704ins5, which were the most common in Korea, which is geographically situated midway between China and Japan, were some of the most common mutations in China and Japan, respectively. These results are especially worthy of notice.

**Electronic supplementary material:**

The online version of this article (doi:10.1186/s12881-016-0319-x) contains supplementary material, which is available to authorized users.

## Background

Mucopolysaccharidosis I (MPS I) is an autosomal recessive lysosomal storage disorder caused by a lack of the lysosomal enzyme α-L-iduronidase (IDUA) [1]. IDUA is an enzyme involved in the metabolism of glycosaminoglycans (GAGs). In the case of IDUA deficiency, heparan sulfate and dermatan sulfate, which are types of GAGs, accumulate in various tissues [2]. MPS I is divided into three phenotypes clinically. The most severe phenotype is Hurler syndrome (MPS IH; MIM#607914), and the mildest phenotype is Scheie syndrome (MPS IS; MIM#607016). The intermediate phenotype is Hurler-Scheie syndrome (MPS IH/S; MIM#607015) [1, 3]. The α-L-iduronidase-coding gene is *IDUA*, which is located in chromosome 4p16.3 [4]. To date, more than 200 *IDUA* mutations have been reported [5]. Of these, missense/nonsense mutations have the highest rates (58 %), followed by splice site mutations (15.9 %), deletions (14 %), and insertions (7.3 %) [5]. The authors first reported *IDUA* mutations in 10 unrelated Korean patients with MPS I in Korea in 2004 [6]. After that, seven patients with MPS I were newly diagnosed, and five novel mutations were confirmed through the *IDUA* mutation analysis in Korea. Thus, the authors report the results of *IDUA* mutations in seven patients and analyzed the *IDUA* mutational spectrum in Korean patients with MPS I, including previous data.

## Methods

### Patients

Seven patients who were diagnosed with MPS I through *IDUA* mutation analysis in the Department of Pediatrics, Samsung Medical Center, from 2009 to 2014 were selected as study subjects in this study. All of these patients were biochemically diagnosed with MPS I because they showed an increase in urinary GAGs and a reduction in IDUA activity in the leukocytes. The urinary GAG levels and IDUA activity for each patient are shown in the Additional file 1: Table S1. All seven patients were unrelated. Depending on the severity of the patients’ clinical symptoms, they were classified as having Hurler, Hurler-Scheie, or Scheie syndrome [1]. The clinical characteristics of patients are shown in the Additional file 1: Table S2. A written informed consent was obtained from each patient or responsible family member. Two patients showed homozygosity for one variant in the *IDUA* mutation analysis. Therefore, we performed array comparative genomic hybridization (CGH) to exclude large deletion in these patients.

### Molecular analysis

Human genomic DNA was extracted from white blood cells using a Wizard genomic DNA purification kit (Promega, Madison, WI). All exons and the flanking regions of the *IDUA* gene were amplified by polymerase chain reaction (PCR) using primers designed by the authors (sequences available upon request) with a thermal cycler (Model 970; Applied Biosystems, Foster City, CA). Direct sequencing of the DNA was performed using the ABI Prism 3100 Genetic Analyzer (Applied Biosystems) with the BigDye Terminator Cycle Sequencing-Ready Reaction Kit (Applied Biosystems). Nucleotides were numbered from the first adenine of the ATG translation initiation codon in the *IDUA* cDNA Reference Sequence NM_000203.3.

### Array CGH

Array Affymetrix CytoScan 750K Array Genomic DNA was extracted from peripheral blood leukocytes using a QIAamp blood mini kit (Qiagen GmbH, Hilden, Germany) according to the manufacturer’s instructions. The microarray assay was performed according to the manufacturer’s protocols (Affymetrix Inc., Santa Clara, CA) [7]. Affymetrix CEL files were analyzed using the Affymetrix Chromosome Analysis Suite (CHAS) version 1.2.2. The reference model file was provided by Affymetrix. The quality control (QC) parameters used in our analyses and their definitions and values, which were recommended by Affymetrix, were as follows: Single nucleotide polymorphism quality control (SNPQC: How well the A and B allele can be resolved) ≥15, median of the absolute values of all pairwise differences (MAPD: How similar the signal distribution of the sample is relative to the reference model file) ≤0.25, and waviness-SD (a global measure of variation of microarray probes that is insensitive to short-range variation and focuses on long-range variation) ≤0.12.

## Results

The *IDUA* mutations of all 14 alleles were identified in a total of seven patients. The genotypes and clinical phenotypes of seven patients are shown in Table 1. In this study, a total of 11 *IDUA* mutations—that is, five missense mutations (p.A79V, p.L346R, p.T388K, p.P496R, and p.C577Y), two nonsense mutations (p.Y618* and p.R628*), two deletions (c.683delC and c.1591delC), one splice site mutation (c.972+1G>A), and one duplication (c.613_617dup) were identified. Among these, p.T388K, p.C577Y, c.683delC, c.1591delC, and c.972+1G>A were variations that had not been reported previously. These five variations were considered novel mutations because they were not observed in an in-house exome database (*n* = 192 individuals). In addition, array CGH was performed in two patients (patient two and patient seven in Table 1) with the homozygosity for one variant to exclude large deletion. The patient two who had a homozygosity for p.T388K showed a loss of heterozygosity of chromosome four. This means the patient two had uniparental disomy of p.T388K mutation in the *IDUA* gene. The patient seven who had a homozygosity for p.L346R showed a normal result in the array CGH.

**Table 1:** Clinical phenotypes and genotypes in 17 unrelated Korean MPS I patients

Pt.	Phenotype	Allele 1	Allele 2	Ref.
1	H	c.613_617dup (p.E207Afs*29)	c.683delC (p.P228Hfs*6)	This report
2	H-S	**c.1163C>A (p.T388K)**	**c.1163C>A (p.T388K)**	This report
3	H-S	c.613_617dup (p.E207Afs*29)	c.1591delC (p.R531Gfs*29)	This report
4	H-S	c.236C>T (p.A79V)	c.1882C>T (p.R628*)	This report
5	H-S	**c.972+1G>A**	**c.1730G>A (p.C577Y)**	This report
6	H-S	c.1487C>G (p.P496R)	c.1854C>A (p.Y618*)	This report
7	S	c.1037T>G (p.L346R)	c.1037T>G (p.L346R)	This report
8	H	?	c.1037T>G (p.L346R)	Ref#6
9	H	c.704ins5 (p.W235Cfs*84)	c.1037T>G (p.L346R)	Ref#6
10	H	c.704ins5 (p.W235Cfs*84)	c.1854C>A (p.Y618*)	Ref#6
11	H	c.704ins5 (p.W235Cfs*84)	c.1037T>G (p.L346R)	Ref#6
12	H	c.704ins5 (p.W235Cfs*84)	c.1037T>G (p.L346R)	Ref#6
13	H	?	c. 193delT	Ref#6
14	H-S	?	c.1037T>G (p.L346R)	Ref#6
15	H-S	?	c.1037T>G (p.L346R)	Ref#6
16	S	?	c.683C>A (p.P228Q)	Ref#6
17	S	c.265C>T (p.A89W)	c.1601C>A (p.S534*)	Ref#6

*H* Hurler syndrome, *H-S* Hurler-Scheie syndrome, *S* Scheie syndrome, *Pt.* patient, *Ref.* reference; Novel mutations are in bold., *?* not yet detected

No one had the same genotype in the seven patients. The homozygosity for p.T388K showed the phenotype of Hurler-Scheie syndrome, and that for p.L346R showed that of Scheie syndrome. The *IDUA* mutations of 10 Korean patients with MPS I that have been previously reported are also shown in Table 1. The results of both this study and the previous study were analyzed together. *IDUA* mutation analysis was performed in a total of 17 unrelated Korean patients with MPS I, and *IDUA* mutations were identified in 29 alleles. There were a total of 16 types of identified *IDUA* mutations as follows: seven missense mutations (p.A79V, p.A89W, p.P228Q, p.L346R, p.T388K, p.P496R, and p.C577Y), three nonsense mutations (p.S534*, p.Y618*, and p.R628*), three deletions (c.193delT, c.683delC, and c.1591delC), one splice site mutation (c.972+1G>A), one duplication (c.613_617dup), and one insertion (c.704ins5). According to the allelic frequency, among a total of 29 alleles, eight were p.L346R (27.6 %), four were c.704ins5 (13.8 %), two were p.Y618* (6.9 %), and two were c.613_617dup (6.9 %). Thus, p.L346R and c.704ins5 mutations accounted for 41.4 % of the entire mutant *IDUA* alleles in Korean patients with MPS I.

## Discussion

This study includes data that shows all of the *IDUA* mutation results of Korean patients with MPS I to the present. Sixteen kinds of mutations were confirmed in 17 patients, and the variety of *IDUA* mutations in MPS I patients could be confirmed. Only p.L346R, c.704ins5, p.Y618*, and c.613_617dup mutations were recurrent mutations, and other mutations were identified only in individual patients. Among these, p.L346R and c.704ins5 accounted for 27.6 and 13.8 %, respectively, in 29 mutant alleles, which accounted for 41.4 % of mutant *IDUA* alleles in Korean patients with MPS I. In particular, three patients showed the same genotype in a total of 17 patients, all of them had heterozygosity for p.L346R and c.704ins5, and they showed the phenotype of Hurler syndrome.

p.L346R has an especially high frequency of mutations in China. Among the mutant *IDUA* alleles of 57 Chinese patients with MPS I, p.A79V accounted for 16.7 % (19/114) and p.L346R accounted for 12.3 % (14/114). These two were most commonly found in Chinese patients with MPS I (Table 2) [8]. Among 57 patients, all three patients who had homozygosity for p.L346R showed the phenotype of Hurler-Scheie syndrome. Thus, it is considered that p.L346R had residual IDUA activity. In this study, only one patient had homozygosity for p.L346R and showed the phenotype of Scheie syndrome. Thus, it is thought that racial difference and polymorphism affected the expression of the mutation.

**Table 2:** Comparison of the common *IDUA* mutations in Korea, China, and Japan

Country	The most common mutation (^a^)	The second common mutation (^a^)
Korea	p.L346R (27.6 %, 8/29)	c.704ins5 (13.8 %, 4/29)
China^b^	p.A79V (16.7 %, 19/114)	p.L346R (12.3 %, 14/114)
Japan^c^	p.R89Q (24 %, 9/38)	c.704ins5 (18 %, 7/38)

^a^, allelic frequency; ^b^[8]; ^c^[9]

c.704ins5 is a mutation that was found only in Japanese patients before *IDUA* mutations in Korean patients with MPS I were reported [9]. In one study aimed at 19 patients, p.R89Q accounted for 24 % (9/38) and c.704ins5 accounted for 18 % (7/38) among mutant alleles. Thus, these two are the most common mutations in Japanese patients with MPS I (Table 2). Patients who had homozygosity for c.704ins5 were associated with Hurler syndrome, and patients who had homozygosity for p.R89Q were associated with Scheie syndrome. Additionally, those who had heterozygosity for c.704ins5 and p.R89Q were associated with Hurler-Scheie syndrome [9]. In this study, there were four patients who had heterozygosity for c.704ins5 and other mutations. All of them showed the phenotype of Hurler syndrome, so it is thought that the c.704ins5 mutation is associated with a severe phenotype. It is interesting that p.L346R and c.704ins5, which are the most common mutations in Korea, which is situated midway between China and Japan, are the most common mutations in China and Japan, respectively. p.A79V, which is common in China, was found in one Korean patient with MPS I. p.R89Q, which is common in Japan, was not found in Korean patients with MPS I. These results indicate that the population in these three countries started from one population and the difference in the types and frequency of the common mutations in these three countries was because of the founder effect. However, a haplotype analysis for their population might be needed in order to prove this.

To date, more than 200 *IDUA* mutations have been reported to the Human Gene Mutation Database [5]. Most mutations occur only within the family. There are a few types of mutations with a high frequency globally and by regional group [10–14]. In Europe, p.W402* and p.Q70*, which are two nonsense mutations, accounted for 37 % (34/92) and 35 % (32/92), respectively, of mutant alleles and are most commonly found [11]. There was a difference in the frequency of each country. p.W402* was the most common, with 38.8 % (14/36) in Spain [10], and p.Q70* was the most common, with 39 % (18/46) and 62 % (21/34), respectively, in Poland and Scandinavia [10, 11]. Additionally, p.W402* and p.Q70* accounted for 39 % (17/44) and 30 % (13/44), respectively, in the United States and showed the highest frequency [15]. p.W402* and p.Q70* created a premature stop codon and showed a severe phenotype in homozygotes [11, 16, 17]. p.W402* and p.Q70*, which are common in Caucasian patients, were not found in Korean patients with MPS I. There have also been no reports of these mutations in China and Japan.

## Conclusions

In this study, the *IDUA* mutational spectrum of Korean patients with MPS I was analyzed, and it was found that p.L346R and c.704ins5 were common mutations in Korea. Furthermore, five novel mutations that were not reported before, p.T388K, p.C577Y, c.683delC, c.1591delC, and c.972+1G>A, were newly discovered. A variety of *IDUA* mutations were able to be identified. It is thought that if the *IDUA* mutation analysis results of newly diagnosed MPS I patients are accumulated, they would help to ascertain the prognosis of the patients by revealing the association between genotype and phenotype and would be very helpful for prenatal diagnosis.

## Abbreviations

CGH, comparative genomic hybridization; GAGs, glycosaminoglycans; IDUA, α-L-iduronidase; MPS I, mucopolysaccharidosis I; MPS IH, Hurler syndrome; MPS IH/S, Hurler-Scheie syndrome; MPS IS, Scheie syndrome

## Additional file

Additional file 1:
**Table S1.** Leukocyte IDUA activity and urinary GAGs of patients with MPS I. **Table S2.** Clinical characteristics of patients with MPS I. (DOCX 15 kb)
