# Supplementary material for: Report of 5 novel mutations of the α-L-iduronidase gene and comparison of Korean mutations in relation with those of Japan or China in patients with mucopolysaccharidosis I
Source: BMC Med Genet. 2016 Aug 12;17:58. doi: 10.1186/s12881-016-0319-x (PMC4983032; doi:10.1186/s12881-016-0319-x)
Supplement: Additional file 1: — Table S1. Leukocyte IDUA activity and urinary GAGs of patients with MPS I. Table S2. Clinical characteristics of patients with MPS I. (DOCX 15 kb) [file 12881_2016_319_MOESM1_ESM.docx]

Table S1 Leukocyte IDUA activity and urinary GAGs of patients with MPS I

| Pt. | Phenotype | IDUA activity^a^ | Urine GAGs^b^ |
| --- | --- | --- | --- |
| 1 | H | 1.92 | 1115.4 |
| 2 | H-S | 2.8 | 733.8 |
| 3 | H-S | 5.9 | 2439.5 |
| 4 | H-S | 6.5 | 202.8 |
| 5 | H-S | <1.0 | 430.3 |
| 6 | H-S | 1.78 | 2517.8 |
| 7 | S | 2.35 | 1011.7 |

H, Hurler syndrome; H-S, Hurler-Scheie syndrome; S, Scheie syndrome; Pt., patient; IDUA, enzyme α-L-iduronidase; GAGs, glycosaminoglycans; ^a^, nmol/mg protein/hr; ^b^, CPC unit/g creatinine

Table S2 Clinical characteristics of patients with MPS I

| Pt. | Phenotype | Age at  diagnosis | Coarse facies | Corneal clouding | Mental retardation | Growth retardation | Hernias | Skeletal abnormalities | Cardiac abnormalities | Hepato-splenomegaly |
| --- | --- | --- | --- | --- | --- | --- | --- | --- | --- | --- |
| 1 | H | 2y3m | **+** | **+** | **+** |  |  | **+** | **+** | **+** |
| 2 | H-S | 1y | **+** | **+** |  |  |  | **+** | **+** | **+** |
| 3 | H-S | 2y | **+** | **+** |  |  |  | **+** |  |  |
| 4 | H-S | 2y7m | **+** | **+** |  |  | **+** | **+** | **+** | **+** |
| 5 | H-S | 33y | **+** | **+** |  | **+** |  | **+** | **+** | **+** |
| 6 | H-S | 1y | **+** | **+** | **+** |  | **+** | **+** | **+** | **+** |
| 7 | S | 2y11m | **+** | **+** |  |  |  | **+** | **+** | **+** |

H, Hurler syndrome; H-S, Hurler-Scheie syndrome; S, Scheie syndrome; Pt., patient; +, Symptoms are present
